# Early generation of a precursor CD8 T cell that can adapt to acute or chronic viral infection

**DOI:** 10.21203/rs.3.rs-3922168/v1

**Published:** 2024-02-12

**Authors:** Daniel T. McManus, Rajesh M. Valanparambil, Christopher B. Medina, Yinghong Hu, Christopher D. Scharer, Ewelina Sobierajska, Daniel Y. Chang, Andreas Wieland, Judong Lee, Tahseen H. Nasti, Masao Hashimoto, James L. Ross, Nataliya Prokhnevska, Maria A. Cardenas, Amanda L. Gill, Elisa C. Clark, Kathleen Abadie, Hao Yuan Kueh, Jonathan Kaye, Byron B. Au-Yeung, Haydn T. Kissick, Rafi Ahmed

**Affiliations:** 1 Emory Vaccine Center, Emory University School of Medicine, Atlanta, GA, USA; 2 Department of Microbiology and Immunology, Emory University School of Medicine, Atlanta, GA, USA; 3 Winship Cancer Institute of Emory University, Atlanta, GA, USA; 4 Department of Urology, Emory University School of Medicine, Atlanta, GA, USA; 5 Department of Otolaryngology, The Ohio State University College of Medicine, Columbus, OH; 6 The Precision Immunology Institute, Icahn School of Medicine at Mount Sinai, New York, NY, USA; 7 Research Division of Immunology, Department of Biomedical Sciences, Cedars-Sinai Medical Center, Los Angeles, CA, USA; 8 Department of Bioengineering, University of Washington, Seattle, WA, USA; 9 Department of Pathology, Mass General Brigham, Harvard Medical School, Boston, MA, USA; 10 Division of Immunology, Lowance Center for Human Immunology, Department of Medicine, Emory University, Atlanta, GA; 11 These authors contributed equally

## Abstract

Virus specific PD-1+ TCF-1+ TOX+ stem-like CD8+ T cells are essential for maintaining T cell responses during chronic infection and are also critical for PD-1 directed immunotherapy. In this study we have used the mouse model of chronic LCMV infection to examine when these virus specific stem-like CD8+ T cells are generated during the course of chronic infection and what is the role of antigen in maintaining the stem-like program. We found that these stem-like CD8+ T cells are generated early (day 5) during chronic infection and that antigen is essential for maintaining their stem-like program. This early generation of stem-like CD8+ T cells suggested that the fate commitment to this cell population was agnostic to the eventual outcome of infection and the immune system prepares *a priori* for a potential chronic infection. Indeed, we found that an identical virus specific stem-cell like CD8+ T cell population was also generated during acute LCMV infection but these cells were lost once the virus was cleared. To determine the fate of these early PD-1+TCF-1+TOX+ stem-like CD8+ T cells that are generated during both acute and chronic LCMV infection we set up two reciprocal adoptive transfer experiments. In the first experiment we transferred day 5 stem-like CD8+ T cells from chronically infected into acutely infected mice and examined their differentiation after viral clearance. We found that these early stem-like CD8+ T cells downregulated canonical markers of the chronic stem-like CD8+ T cells and expressed markers (CD127 and CD62L) associated with central memory CD8+ T cells. In the second experiment, we transferred day 5 stem-like cells from acutely infected mice into chronically infected mice and found that these CD8+ T cells could function like resource cells after transfer into a chronic environment by generating effector CD8+ T cells in both lymphoid and non-lymphoid tissues while also maintaining the number of stem-like CD8+ T cells. These findings provide insight into the generation and maintenance of virus specific stem-like CD8+ T cells that play a critical role in chronic viral infection. In particular, our study highlights the early generation of stem-like CD8+ T cells and their ability to adapt to either an acute or chronic infection. These findings are of broad significance since these novel stem-like CD8+ T cells play an important role in not only viral infections but also in cancer and autoimmunity.

The importance of PD-1+ TCF-1+ stem-like CD8+ T cells is now well established in chronic viral infections and cancer^1-9^. These pluripotent CD8+ T cells can undergo a slow self-renewal and also differentiate into effector- like cells that eventually get terminally differentiated /exhausted (TD) ^1,2,10-12^. These stem-like CD8+ T cells, also referred to as Tpex (precursors of exhausted T cells), are critical for maintaining the CD8+ T cell response under conditions of chronic antigen stimulation ^13-16^. This is the key resource cell that maintains the on-going CD8+ T cell response. Importantly, these are also the cells that respond to PD-1 blockade by increased proliferation and differentiation resulting in the generation of more effector-like CD8+ T cells^1,17,18^. The chronic stem-like CD8+ T cells are transcriptionally and epigenetically distinct from the more differentiated effector-like and exhausted CD8+ T cells and interestingly, they are also distinct from effector and memory CD8+ T cells generated after an acute viral infection^1,10,19-21^. A novel feature of the chronic PD-1+ TCF-1+ stem-like CD8+ T cells is that they have captured certain aspects of CD4+ T follicular helper (Tfh) cells and express Bcl-6 and CXCR5 and do not express effector molecules such as granzyme B and perforin^1^.

In this study we have addressed several fundamental questions about the generation, maintenance, and differentiation of antigen specific stem-like CD8+ T cells using the mouse model of chronic lymphocytic choriomeningitis virus (LCMV) infection^22^. We have asked the following questions: 1. When are these stem-like CD8+ T cells generated during the course of chronic viral infection? How does their program change over time? What is the role of antigen in maintaining the stem-like program? How does the differentiation trajectory of the virus specific PD-1+ TCF-1+ CD8+ T cells change in the presence or absence of antigen?

## Results and Discussion

### Early generation of virus specific stem-like CD8+ T cells during chronic infection

The first question we addressed was when are the PD-1+ TCF-1+ stem-like CD8+ T cells generated during the course of chronic LCMV infection and how do these cells compare with the stem-like CD8+ T cells present during the later established phases of chronic infection. We have used the LCMV clone 13 strain to study chronic infection^22^. Our previous studies have shown that PD-1+ TCF-1+ Tim-3− virus specific CD8+ T cells are detectable in the spleens of mice during the first week of chronic infection^1,23^. Other studies have also reported similar observations^2,24-27^. To extend these observations we did a detailed comparison of LCMV specific stem-like CD8+ T cells present at day 5 versus day 45 post-infection at the phenotypic, anatomical, transcriptional, and epigenetic level.

**Figure 1:**
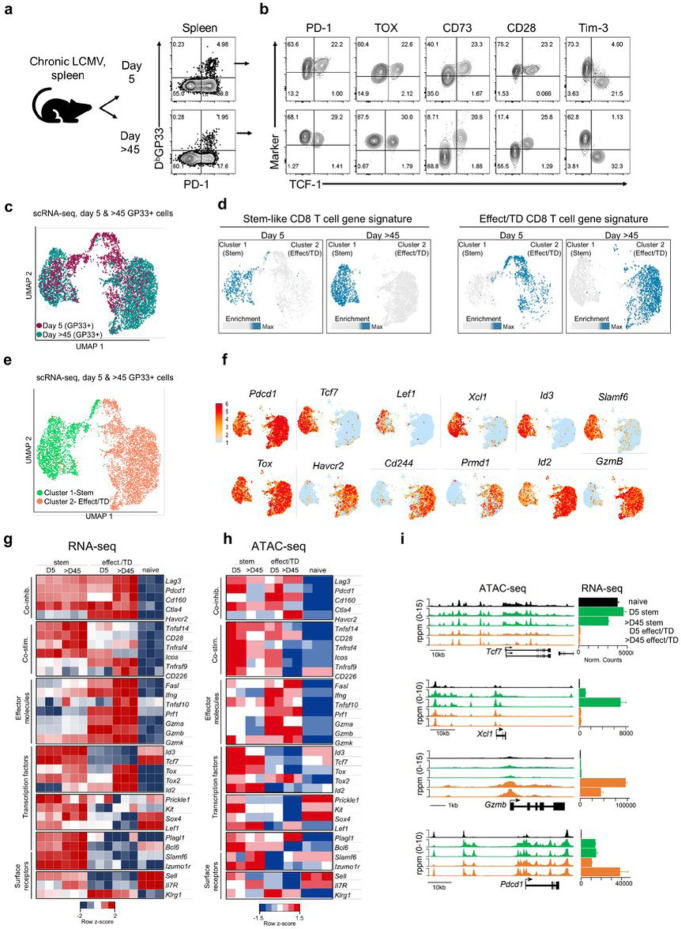
Early fate decision of stem-like CD8+ T cells during chronic infection. Mice were infected with LCMV clone 13 after transient CD4+ T cell depletion which results in high viral titers and life-long viral infection (Matloubian et al., 1994). a,b, Phenotypic analysis of LCMV-specific DbGP33+ CD8+ T cells in the spleen on days 5 and >45 after chronic infection. c-f, scRNA-seq analyses of splenic DbGP33+ CD8+ T cells on days 5 (60,000 cells) and >45 (40,000 cells) after chronic infection. c, Combined data from day 5 and >45 cells projected as a UMAP plot. d, day 5 and >45 cells formed two distinct clusters based on enrichment for the stem-like (cluster 1) versus effector/terminally differentiated (effect./TD) (cluster 2) CD8+ T cell gene signatures (GSE84105, Im et al., 2016). Max = maximum. e,f, UMAP plots showing expression of selected genes by each cluster. g-i, Stem-like (CD73+ Tim-3−) and effect./TD (CD73− Tim-3+) CD8+ T cells were sorted from day 5 and >45 spleens for RNA- and ATAC-seq analysis. Bulk PD-1+ cells were used for RNA-seq whereas LCMV GP33-specific P14 cells were used for ATAC-seq. g,h, Heatmaps showing relative gene expression (g) and accessibility (h) of selected genes. i, Accessibility tracks (ATAC-seq, left) and normalized counts (RNA-seq, right) for selected genes. RNA- and ATAC-seq results are each from 1 experiment.

Flow cytometric analysis of LCMV specific (H-2D^b^ GP33) CD8+ T cells in the spleen revealed a subset of virus specific cells expressing TCF-1 as early as day 5 after LCMV clone 13 infection (Fig. 1a and b). In addition to TCF-1, these day 5 cells shared other markers with *bona fide* stem-like CD8+ T cells from the late phase of chronic infection (>d45); they expressed PD-1, TOX, CD73, and CD28 and were negative for Tim-3 (Fig. 1b). A key characteristic of stem-like CD8+ T cells in the spleen is their anatomical bias for the white pulp region whereas effect./TD cells are located mainly in the red pulp area^1^. To determine when this spatial organization begins, we used intravascular labeling^28,29^ to compare the intrasplenic locations of GP33-specific TCF-1+ Tim-3-stemlike and TCF-1− Tim-3+ effect./TD cells on days 5 and >45 (Extended Data Fig. 1a). Greater than 50% of GP33-specific stem-like (TCF-1+ Tim-3−) CD8+ T cells were located in the blood-inaccessible white pulp (i.v. label negative) on day 5 compared to just 10% of GP33-specific effect./TD (TCF-1− Tim-3+) CD8+ T cells. The frequency of stem-like CD8+ T cells in the white pulp increased to >80% between days 5 and >45, whereas the bias of effect./TD cells for the red pulp (i.v. staining positive) remained throughout infection (Extended Data Fig. 1b and c). Imaging of PD-1+ stem-like and effect./TD CD8+ T cells confirmed our i.v. labeling results showing a greater percentage (~1.5-2 fold) of stem-like compared to effect./TD CD8+ T cells in the white pulp on day 5 (Extended Data Fig. 1d and e). Thus, the anatomical bias of stem-like CD8+ T cells for the white pulp region begins early during chronic infection.

We next performed single cell RNA-sequencing (scRNA-seq) of GP33-specific cells from days 5 and >45 after chronic infection to determine if the transcriptional signature of stem-like CD8+ T cells could be detected at the day 5 time point (Extended Data Fig. 2a). Interestingly, day 5 and >45 cells formed two major clusters unrelated to time of infection (Fig. 1c). Cluster 1 cells enriched for the stem-like CD8+ T cell gene signature whereas cluster 2 cells enriched for an effect./TD gene signature (Fig. 1d and Extended Data Fig. 2b and c)^1^. Cells in both clusters expressed *Pdcd1* and *Tox.* Cluster 1 (Stem) cells expressed high levels of *Tcf7* as well as two transcription factors associated with stem-like CD8+ T cells, *Lef1* and *Id3*^1,24^. They also expressed *Xcl1* and *Slamf6,* two additional markers unique to stem-like CD8+ T cells during chronic infection (Fig. 1e, f, and Extended Data Fig. 2d and e)^1,10^. In contrast, co-inhibitory receptors *Havcr2* and *Cd244,* transcription factors associated with effector differentiation, *Prdm1* and *Id2* and the cytotoxic molecule, *Gzmb,* were almost exclusively expressed by cells in cluster 2 (effect./TD) (Fig. 1e, f, and Extended Data Fig. 2f and g) ^30-33^.

To further complement the scRNA-seq data we performed transcriptional (RNA-seq) and epigenetic (ATAC-seq) analysis of sorted stem-like (PD-1+ CD73+ Tim-3−) and more differentiated effector/TD (PD-1+ CD73− Tim-3+) subsets at days 5 and >45 post-infection. Endogenous GP33-specific stem-like and effect./TD were used for RNA-seq (Extended Data Fig. 3a) whereas monoclonal TCR transgenic GP33-specific CD8 T cell (P14s) subsets were used for ATAC-seq (Extended Data Fig. 3b). Stem-like and effect./TD subsets from both time points shared in the expression and accessibility of multiple inhibitory molecules (*Lag3, Pdcd1, Cd160, Ctla4*) (Fig. 1g-i). Interestingly, many of the gene expression and chromatin accessibility hallmarks of canonical stem-like CD8+ T cells were in place as early as day 5^1^. These included higher expression and accessibility for costimulatory molecules like *Tnfsf14, Tnfrsf4,* and most notably *Cd28,* which is not only required for the response to PD-1 blockade but is also an important regulator of stem-like CD8+ T cell self-renewal and differentiation^34,35^. Gene expression and accessibility for effector genes (*Fas1, Ifng, Prf1, Gzma, Gzmb*) were low for stemlike relative to effect./TD CD8+ T cells, even on day 5 when antigen and inflammation levels are high (Fig. 1g-i). This highlights a defining feature of the stem-like CD8 T cell program: active repression at key effector loci regardless of an effector-favored environment. Reciprocally, high expression and accessibility for transcription factors associated with longevity and self-renewal in T cells (*Id3, Tcf7, Prickle1, Kit, Sox4, Lef*) and CD4+ Tfh molecules (*Bcl-6, Slamf6, Izumo1r*) was also observed in day 5 stem-like CD8 T cells (Fig. 1g-i) ^36-39^.

While early and late stem-like CD8+ T cells are similar, long-term antigen exposure also leads to substantial transcriptional, epigenetic, and metabolic changes to T cells^19,40-42^. Consistent with this, day 5 and >45 stem-like CD8+ T cells differed in the accessibility of 10684 regions (DARs) and expression of 3643 genes (DEGs) (Extended Data Fig. 4a and b). Genes that were enriched in day 5 stem-like CD8+ T cells were related to amino acid metabolism, glucose metabolism, mTOR signaling, and the TCA cycle, suggestive of their rapid proliferation at earlier timepoints (Extended Data Fig. 4c and d)^43^. Consistent with these changes, flow cytometry showed that between days 5 to >45, stem-like CD8+ T cells lost expression of the proliferation marker, Ki67, and decreased in cell size (Extended Data Fig. 4e and f). Transforming growth factor β (TGF-β) was recently shown to mediate the quiescence of stem-like CD8+ T cells by regulating the expression of inhibitory molecules like *Cd200r1, Cd200, CD83, Lrig1, Tnfsrf8,* and *Izumo1r*^26,44^. Interestingly, these molecules were highly elevated on day >45 stem-like CD8+ T cells relative to their day 5 counterparts and naïve CD8+ T cells (Extended Data Fig. 4g).

Taken together, the data indicate that virus specific stem-like CD8+ T cells with the canonical transcriptional signature and epigenetic marks of *bona-fide* stem-like CD8+ T cells can be detected as early as 5 days after infection with LCMV clone 13. While there are also important differences between the day 5 and day >45 LCMV specific stem-like CD8+ T cells (Extended Data Fig. 4), the fate decision for generating the stem-like CD8+ T cells is made early after infection.

### Identical populations of stem-like CD8+ T cells are generated early after acute or chronic viral infection

**Figure 2:**
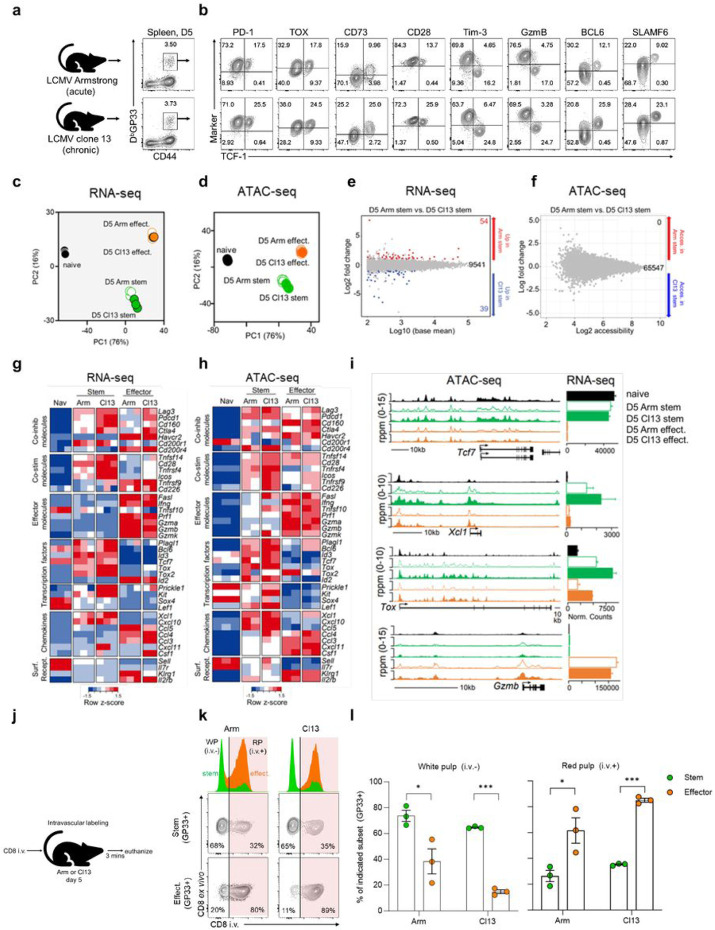
Identical populations of stem-like CD8+ T cells are generated early after acute and chronic infection. a,b, Phenotypic analysis of DbGP33+ CD8+ T cells in the spleen on day 5 after LCMV Armstrong (acute) or clone 13 (chronic) infection. Flow plots are representative of 3 independent experiments (n= 4-5 recipients per donor per experiment). Both infections were administered intravenously (i.v.) at a dose of 2x10^6^ PFU. c-i, 2x10^5^ P14 cells were transferred into congenically distinct naïve mice prior to intravenous infection with LCMV Armstrong or clone 13. LCMV GP33-specific P14 stem-like and effector CD8+ T cells were isolated from the spleens of acute or chronic LCMV mice on day 5 for RNA- and ATAC-seq analysis. c,d, PCA plots showing RNA- (c) and ATAC-seq (d) profiles of indicated subsets. e,f, MA plots showing differentially expressed genes (e) and differentially accessible regions (f) between indicated subsets. Red dots/number = genes upregulated/more accessible in stem-like cells from acute infection. Blue dots/number = genes upregulated/more accessible in stem-like cells from chronic infection. Grey dots/black number = genes not differentially expressed/accessible. g,h, Heatmaps showing relative expression (g) and accessibility (h) of selected genes by indicated subsets. i, Accessibility tracks (ATAC-seq, left) and normalized counts (RNA-seq, right) for selected genes. RNA- and ATAC-seq results are each from 1 independent experiment. j, On day 5 after acute or chronic infection, mice were injected with fluorescently-labeled anti-CD8α (3μg/mouse) and euthanized 3 mins later for flow cytometry analysis. k, Flow plots/histograms showing frequencies of DbGP33+ TCF-1+ Tim-3− (stem-like) and TCF-1-Tim-3 (effect./TD) CD8+ T cells in the white pulp (CD8α i.v. antibody negative) or the red pulp (CD8α i.v. antibody positive). Numbers on flow plots are frequencies. l, Frequencies of stem-like and effect./TD CD8+ T cells in the white pulp (left) and red pulp (right). Flow plots/histograms are representative of 1 of 2 independent experiments (n= 3-5 mice per infection group). Graph shows mean±SEM, data are from 1 of 2 independent experiments (n= 3 per infection group) and p values shown from Wilcoxon matched-pairs signed rank test. * and *** indicate p values of less than 0.05 and 0.001, respectively.

The above experiments show that the chronic resource stem-like CD8+ T cells are generated within a few days after LCMV clone 13 infection. This is a surprising finding since at this early stage the immune system does not know whether this is going to be a persistent infection or an acute infection. Thus, it appears that the stem-like CD8+ T cell fate commitment is agnostic to antigen outcome, implying that the host prepares *a priori* for long-term antigen exposure. To address this experimentally, we examined the phenotype of virus specific CD8+ T cells early after acute (LCMV Armstrong) versus chronic (LCMV clone 13) infection^22^. For a fair comparison, acute and chronic LCMV infections were both performed intravenously at a dose of 2 x 10^6^ plaque forming units (PFU). Interestingly, GP33-specific CD8+ T cells with the stem-like phenotype (PD-1+ TCF-1+) were also present on day 5 after acute LCMV Armstrong infection (Fig. 2a and b). These cells shared multiple markers with day 5 stem-like CD8+ T cells from chronic infection including the expression of CD73, CD4+ Tfh markers (Bcl-6 and Slamf6), costimulatory molecules (CD27, CD28, and CD226), and the chemokine receptor, CXCR3 (Fig. 2b and Extended Data Fig. 5a). Moreover, in both infections these day 5 stem-like cells did not express effector markers like Tim-3, GzmB, CX3CR1, KLRG1, and CD25 (Fig. 2b and Extended Data Fig. 5a). Of note, PD-1+ TCF-1+ cells were present at a lower frequency in acute infection but absolute numbers of these cells did not differ from chronic infection (Extended Data Fig. 5b and c). Thus, antigen-specific CD8+ T cells with the same phenotype as stem-like CD8+ T cells from chronic infection are generated during acute infection.

For a more detailed analysis we compared gene expression (RNA-seq) and chromatin accessibility (ATAC-seq) in stem-like CD8+ T cells sorted on day 5 after acute versus chronic infection (Extended Data Fig. 5d). When projected onto principal component analysis (PCA) plots, stem-like CD8+ T cells from acute and chronic infection were positioned in almost the same location for both RNA- and ATAC-seq analyses (Fig. 2c and d). They were remarkably similar, differing in gene expression by less than 1% (DEGs= 93/9634) and displaying no differentially accessible chromatin regions (DARs = 0/65547) (Fig. 2e and f). Irrespective of infection origin, costimulatory molecules (*Cd28, Tnfrsf4, Icos*) were more highly expressed and accessible in stem-like relative to effector CD8+ T cells. They showed low expression/accessibility at effector loci (*Fas1, Ifng, Prf1, Gzma, Gzmb*) but higher expression/accessibility for CD4+ Tfh transcription factors (*Plagl1, Bcl-6*), long-lived memory cells (*Tcf7, Id3*), and chemokines expressed by stem-like CD8+ T cells (*Xcl1, Cxcl10*) (Fig. 2g-i) ^45^.

We next asked if the early anatomical divergence of stem-like and effector CD8+ T cells observed during the early stages of chronic infection (Extended Data Fig. 1) also happens during acute infection. Intravascular labeling was performed on mice 5 days after acute or chronic LCMV infection to determine the intrasplenic locations of GP33-specific stem-like and effector CD8+ T cells (Fig. 2j). Regardless of infection, greater than 60% of GP33-specific stem-like CD8 T cells were localized to the white pulp compared to less than 20% of effectors (Fig. 2k and l). Thus, the anatomical organization of virus-specific stem and effector CD8+ T cells occurs early during both acute and chronic infection.

**Figure 3:**
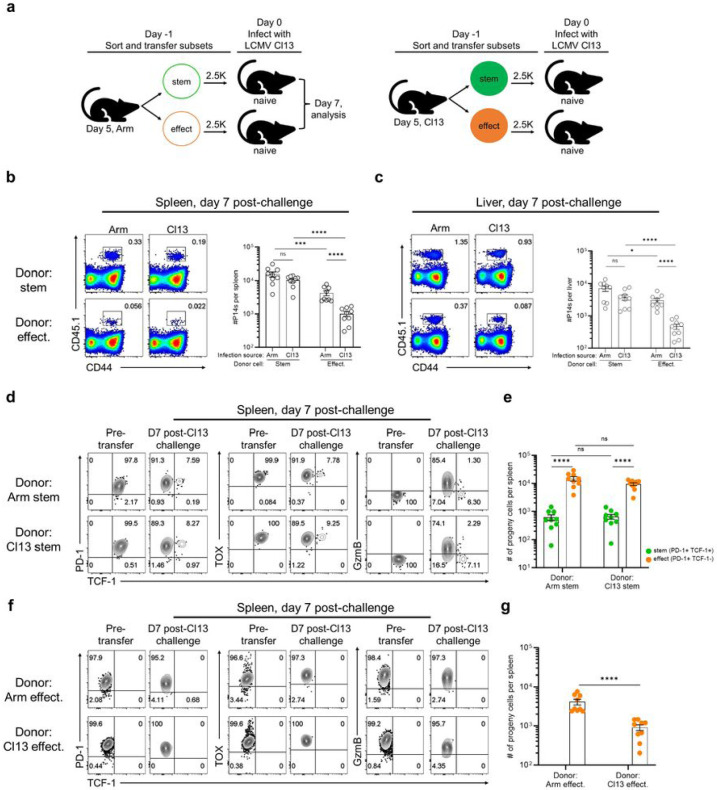
Stem-like CD8+ T cells from acute and chronic infection are equally able to expand, self-renew, and effector differentiate after clone 13 challenge. a, CD45.1+ LCMV GP33-specific P14 stem and effect. CD8+ T cells were isolated from LCMV Arm or Cl13-infected spleens on day 5 and transferred into naïve CD45.2+ mice. Recipients were then challenged i.v. with 2x106 PFU of LCMV Cl13. b,c, Frequency and numbers of donor cells recovered from indicated recipient spleens (b) and livers (c) on day 7 after clone 13 challenge. d, Expression of indicated markers on stem-like donor cells from acute (top) or chronic (bottom) infection pre- and on day 7 post-clone 13 challenge. e, Numbers of splenic stem-like (TCF-1+ GzmB−) and effector (TCF-1− GzmB+) CD8+ T cells derived from indicated donor cells. f, Expression of indicated markers on effector donor cells from acute (top) or chronic (bottom) infection pre- and on day 7 post-clone 13 challenge. g, Numbers of splenic stem-like (TCF-1+ GzmB−) and effector (TCF-1− GzmB+) CD8+ T cells derived from indicated donor cells. Flow plots represent 2 of 3 independent experiments (n= 4-5 recipient mice per group per experiment). Summary graphs show mean ± SEM and data are combined from 2/3 independent experiments (n=9 per group). P values calculated using Mann-Whitney test. *, ***, and **** indicate p values of less than 0.05, 0.001, and 0.0001, respectively.

It was important to also do a functional comparison of the early (day 5) stem-like CD8+ T cells from acute and chronic infection. To do this we examined the ability of stem-like CD8+ T cells from acute versus chronic infection to respond to a LCMV clone 13 challenge. LCMV GP33-specific day 5 P14 stem-like (Slamf6+ Tim-3−) and effector (Slamf6− Tim-3+) CD8+ T cells were isolated from the spleens of acutely or chronically infected mice and then transferred in equal numbers into separate groups of congenically distinct naive mice. Recipients were then challenged with LCMV clone 13 and 7 days later the recovery and phenotype of donor cells were assessed (Fig. 3a and Extended Data Fig. 6a). Higher numbers of donor cells were recovered from the spleens and livers of mice that received stem-like compared to effector CD8+ T cells (Fig. 3b and c). Interestingly, stem-like cells from acute and chronic LCMV gave rise to similar numbers of progeny in both the spleen and liver (Fig. 3b and c). These were primarily effector CD8+ T cells (PD-1+ TCF-1− TOX+ GzmB+) that were generated in similar numbers by the day 5 stem-like CD8+ T cells from either LCMV Armstrong or clone 13 infection (Fig. 3d and e). The remainder of the cells at day 7 post-challenge consisted of stem-like CD8+ T cells (PD-1+ TCF-1+ TOX+ GzmB−) that were also maintained in equal numbers (Fig. 3d and e). In mice that received the effector subset (Slamf6− Tim-3+), there was less expansion of the donor cells (Fig. 3b and c) and these cells did not show any change in their phenotype (Fig. 3f and g). This experiment shows that stem-like CD8+ T cells from acute and chronic LCMV are equally capable of expansion, self-renewal, and effector differentiation in response to clone 13 challenge. Thus, functionally similar populations of stem-like CD8+ T cells are generated during the early phase of acute or chronic LCMV infection.

### Continuous antigen exposure is required for the maintenance of stem-like CD8+ T cells during viral infection

**Figure 4:**
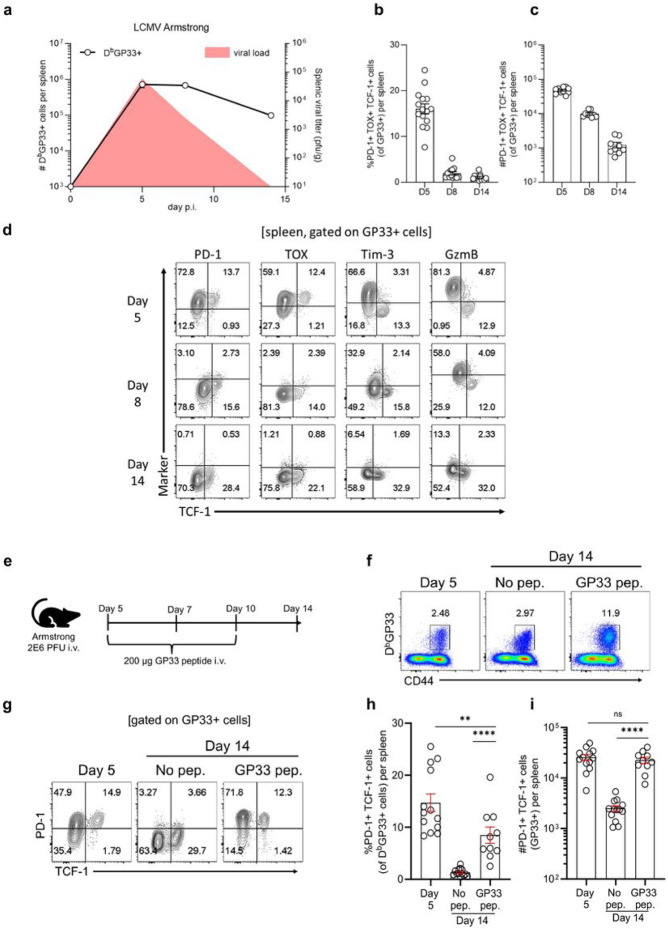
Antigen is required for the maintenance of stem-like CD8+ T cells during acute viral infection. Mice were infected intravenously with 2x10^6^ PFU of LCMV Armstrong (acute). a, Viral titer kinetics (red curve) and numbers of LCMV-specific DbGP33+ CD8+ T cells in the spleen over time. Viral titer data shown are from one independent experiment (n= 3-5 mice per time point) and are consistent with historical data (Matloubian et al., 1993). Curve showing numbers of DbGP33+ CD8+ T cells is combined data from 3 independent experiments (n= 12-15 per time point) with mean and SEM included. b,c, Frequency (b) and numbers (c) of GP33+ PD-1+ TOX+ TCF-1+ stem-like CD8+ T cells in the spleen at indicated times postacute infection. Graphs b and c show mean ± SEM and data are combined from 3 independent experiments (n= 12-15 mice per time point). d, Expression of indicated markers on GP33+TCF-1+ CD8+ T cells during course of acute infection. Flow plots are representative of 1 of 3 independent experiments (n=3-5 mice per time point per experiment). e, On day 5 after acute infection, mice were treated i.v. with 200ug of GP33 peptide on days 5, 7, and 10. After clearance of infection (day 14), the phenotype of DbGP33+ CD8+ T cells was examined f, Frequency of DbGP33+ CD8+ T cells in the spleen. g-i, Frequency and number of DbGP33+PD-1+ TCF-1+ CD8+ T cells in the spleen. Flow plots shown are representative of 1 of 3 independent experiments (n= 3-5 mice per group). Graphs h and i show mean ± SEM and are combined data from 3 independent experiments (n= 10-13 mice per group). P values calculated using Mann-Whitney test. ** and **** indicate p values of less than 0.01, and 0.0001, respectively.

Our results showing that the canonical stem-like CD8+ T cells are also generated during the early stages of acute LCMV infection while antigen is still present raises the obvious question of what happens to these cells as the acute infection is rapidly cleared. To address this question we monitored expression of phenotypic markers that define these LCMV-specific stem-like CD8+ T cells (PD-1+ TOX+ TCF-1+) at days 5, 8 and 14 post-infection with LCMV Armstrong. The data in Fig. 4a-d show that LCMV specific CD8+ T cells of this phenotype are lost as the infection is cleared.

The loss of stem-like CD8+ T cells following clearance of acute infection suggested that continuous antigen exposure might be required for the maintenance of stem-like CD8+ T cells. To test this, we injected acutely infected mice with 200ug of GP33 peptide on days 5, 7, and 10 following infection with the LCMV Armstrong strain (Fig. 4e). This resulted in an increase in the frequency of GP33-specific CD8+ T cells (Fig. 4f and Extended Data Fig. 7a) but more importantly this peptide immunization during acute infection prevented the loss of LCMV GP33-specific stem-like CD8+ T cells (Fig. 4g-i and Extended Data Fig. 7b). Stem-like CD8+ T cells expressing PD-1, TOX, and TCF-1 were maintained at day 14 in numbers equivalent to what was seen at day 5. In contrast, acutely infected mice not injected with peptide showed a significant drop in the number of GP33-specific stem-like CD8+ T cells (Fig. 4g-i). Providing antigen exposure during this interval by peptide also increased the expression of Slamf6 and Bcl-6 (Extended Data Fig.7b). These data highlight the importance of antigen for maintaining the stem-like CD8+ T cell program.

**Figure 5:**
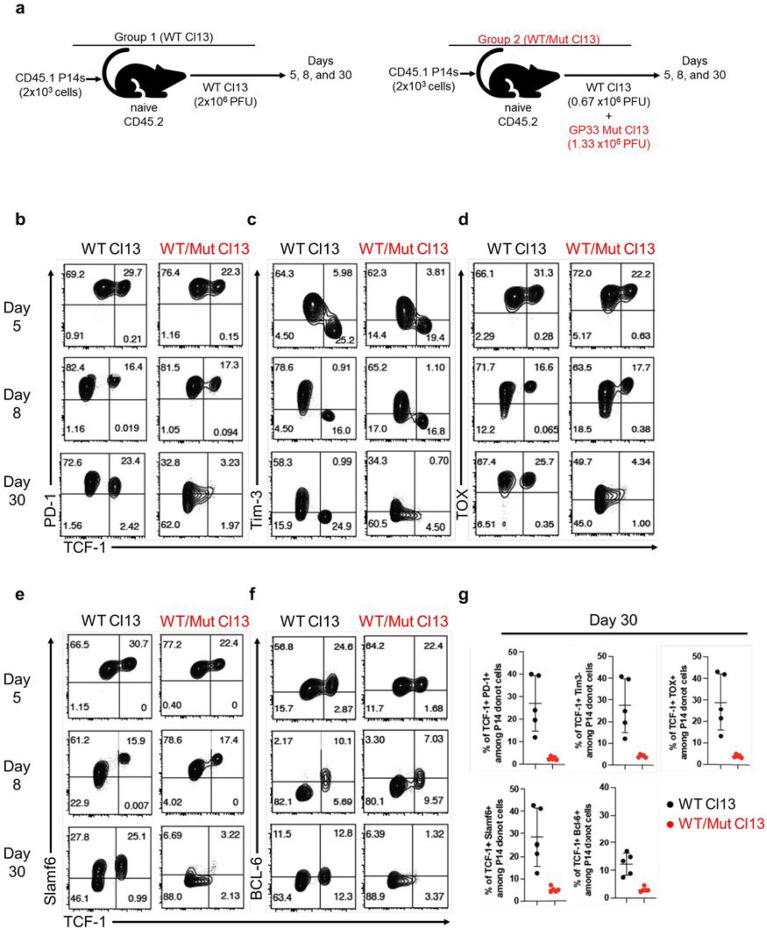
High antigen levels are necessary to sustain stem-like CD8+ T cells during chronic infection. a, 2x10^3^ LCMV GP33-specific CD45.1+ P14 CD8+ T cells were transferred into naïve CD45.2+ mice. Recipient mice were then infected with either 2x10^6^ PFU wildtype LCMV clone 13 (WT Cl13) or with a combined dose of 2x10^6^ PFU consisting of 0.66 x10^6^ PFU WT clone 13 plus 1.33 x10^6^ PFU of a mutant GP33-deficient LCMV clone 13 strain (WT/Mut Cl13). b-f, Flow plots showing expression of indicated markers on splenic LCMV GP33-specific P14 CD8+ T cells from indicated infection groups at various times post-infection. g, Frequencies of indicated splenic TCF-1+ P14 CD8 T cell subsets on day 30 after WT Cl13 or WT/Mut Cl13 infections. Flow plots are representative of 1 of 2 independent experiments (WT Cl13 n=5-10, WT/Mut Cl13 n= 4-5). Graphs show data from one independent experiment (WT Cl13 n=5, WT/Mut Cl13 n= 5).

We next asked how antigen versus the chronic infection environment impacts the maintenance of PD-1+ TCF-1+ stem-like CD8+ T cells during chronic infection. To address this question, we took advantage of a LCMV clone 13 mutant with a mutation in the GP33 epitope (valine to alanine amino acid substitution at position 35) that reduces binding of the peptide to D^b 46,47^. The set-up for this experiment is described in Fig. 5a; one group of mice were infected with WT LCMV clone 13 as our control group and the other group of mice were infected with a mixture of WT LCMV clone 13 and the GP33 mutant clone 13 (2:1 ratio in favor of the mutant virus). As described previously, under these conditions of mixed infection there is initial presentation of GP33 from the WT virus but over time the GP33 mutant virus becomes dominant since there was a higher ratio of the mutant virus in the inoculum but more importantly because there is less efficient killing of cells infected with the mutant strain. Thus, the WT LCMV clone 13 strain gets outcompeted by the LCMV mutant strain and GP33 presentation decreases over time^48,49^. This chronic infection setting enables one to track GP33-specific CD8+ T cells as exposure to their cognate antigen disappears over time due to the dominance of the GP33 mutant virus but the viral burden remains high along with the chronic infection environment. LCMV GP33-specific P14 CD8+ T cells were used in this experiment to track their differentiation under these two different conditions of chronic infection where there was either continuous antigen stimulation (group 1) or gradual loss of antigen stimulation (group 2). Similar frequencies of P14 stem-like (TCF-1+ Tim-3−) and effector (TCF-1 Tim-3+) CD8+ T cells were observed in WT Cl13 and WT/Mut Cl13 infections on days 5 and 8. Also, mixed infection with WT/Mut Cl13 resulted in high expression of PD-1 and TOX on both stem-like and effector-like CD8+ T cells at these early time points (Fig. 5b-d). This is important as it indicates that sufficient levels of GP33 were present for the initial priming, differentiation, and expansion of P14 CD8+ T cells in WT/Mut Cl13-infected mice. The expression of Slamf6 and Bcl-6 was also similar in both groups of mice (Fig. 5e-g). However, by day 30 there was a striking loss of the GP33 (P14) stem-like CD8+ T cells in the mixed WT/Mut Cl13-infected mice, and the expression of PD-1, TOX, Slamf6, and Bcl-6 declined markedly relative to WT Cl13-infected mice (Fig. 5b-g). A critical control in this experiment is that the loss of stem-like CD8+ T cells in the WT/Mut Cl13-infected mice was selective for the GP33 epitope and that LCMV GP276-specific stem-like CD8+ T cells were maintained equally well in WT Cl13-infected mice and the mixed WT/Mut Cl13-infected mice (Extended Data Fig. 8a and b). These results show unequivocally that antigen is essential to maintain the stemlike CD8+ T cell program during chronic infection and that the chronic environment alone cannot do it.

An obvious assumption from the above experiments is that the PD-1+ TCF-1+ stem-like CD8+ T cells are continuously getting TCR signaling during chronic LCMV infection. To provide direct evidence for this we used *Nur77*-GFP reporter mice^50-52^. As shown in Extended Data Fig. 9a-c, nearly all of the PD-1+ GP33-specific CD8+ T cells during chronic infection are *Nur77*-GFP+ and >80% of the stem-like CD8+ T cells are positive for *Nur77*-GFP+. In contrast, GP33-specific memory CD8+ T cells present after clearance of an acute LCMV infection are negative for both *Nur77*-GFP and PD-1. Taken together these results show that antigen stimulation is actively maintaining the novel program of these virus-specific stem-like CD8+ T cells during chronic infection. It is particularly interesting that these PD-1+ *Nur77*+ TCF-1+ CD8+ T cells are getting TCR signaling but they do not express effector molecules like granzyme B and perforin. This highlights the novel CD4+ Tfh aspect of these chronic PD-1+ stem-like CD8+ T cells with high expression of Bcl-6 and an overall transcriptional signature that resembles CD4+ Tfh cells^1^.

### Early day 5 virus-specific stem-like CD8+ T cells from LCMV clone 13-infected mice acquire the phenotype of central memory CD8+ T cells after transfer into acutely infected mice

**Figure 6:**
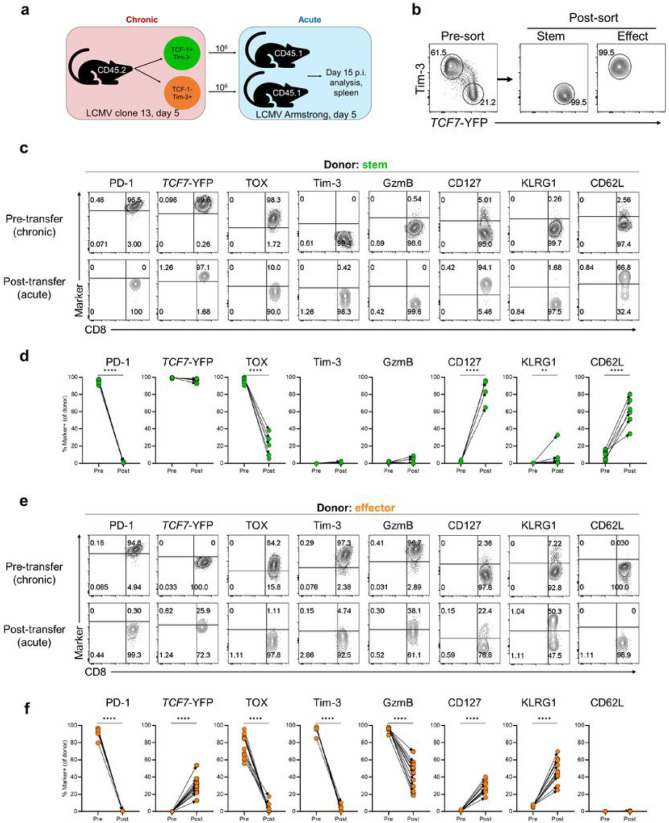
Early stem-like CD8+ T cells from chronic viral infection become central memory CD8+ T cells after transfer into acutely infected mice. a,b 2x10^3^ TCF7-YFP reporter P14 CD8+ T cells were transferred into congenically mismatched naïve mice prior to intravenous infection with 2x10^6^ PFU LCMV clone 13. On day 5 p.i. TCF7-YFP+ Tim-3− (stem) and TCF7-YFP-Tim-3+ (effect.) P14 CD8+ T cells were isolated from the spleen via FACS and transferred intravenously into separate groups of congenically distinct, day 5 LCMV Armstrong-infected recipient mice. 10 days after transfer, the phenotype of donor cells was analyzed. c, Expression of indicated markers on stem-like donor cells pre- (spleen, day 5 p.i., chronic) and post-transfer (day 15 p.i., acute) in the spleen. d, Frequencies of stem-like donor cells positive for indicated markers pre- and post-transfer. e, Expression of indicated markers on effector donor cells pre- and post-transfer in the spleen. f, Frequencies of effector donor cells positive for indicated markers pre- and post-transfer. Flow plots are representative of 1 of 2 independent experiments. Graphs show data pooled from 2 independent experiments (pretransfer n=15, post-transfer n= 6-12). P values calculated using Mann-Whitney test. ** and **** indicate p values of less than 0.01, and 0.0001, respectively.

Our above results show that PD-1+ TCF-1+ stem-like CD8+ T cells are generated early after infection and that continuous antigen exposure is needed to preserve the essential features of these chronic stem-like CD8+ T cells. This raises the important question of what will happen to the day 5 stem-like CD8+ T cells generated during the early phase of chronic infection if they are transferred into mice where antigen is being cleared? How will their differentiation trajectory change in the absence of antigen and what will their progeny cells look like? To address this question, we sorted stem-like and effector P14 cells from clone 13-infected mice at day 5 and transferred these sorted P14 CD8+ T cell subsets into congenically distinct mice acutely infected with LCMV Armstrong at day 5. The fate of these adoptively transferred cells was then analyzed at day 15 post Armstrong infection, a time point when the infection has been fully resolved. We used *Tcf7*-YFP reporter P14 cells in this experiment; stem-like cells were sorted as *Tcf7*-YFP+ Tim-3− and the effector cells as *Tcf7*-YFP− Tim-3+ (Fig. 6a and b). Both donor populations were detectable in the spleens of recipient mice 10 days after transfer and there were more cells recovered in mice that received the effector P14s (Extended Data Fig. 10a and b). The transferred donor stem-like cells retained TCF-1 expression and remained negative for Tim-3 and granzyme B but there were other striking phenotypic changes in these cells; PD-1 and TOX were almost completely downregulated and most interestingly, there was upregulation of CD127 and CD62L. Also of interest is that the donor stem-like cells remained mostly negative for KLRG1 (Fig. 6c and d). Co-staining of the canonical memory T cells markers (CD127, KLRG1, CD62L) showed that the donor stem-like cells from clone 13-infected mice were acquiring markers associated with central memory T cells in the absence of antigen (Extended Data Fig. 10c) ^53-55^. Thus, the virus-specific PD-1+ TCF-1+ CD8+ T cells generated during the early phase of chronic infection can also adapt to an acute infection and contribute to the pool of central memory CD8+ T cells following antigen clearance.

The donor effector cell population from clone 13-infected mice also exhibited several phenotypic changes after transfer into the acutely infected mice (Fig. 6e and f). These cells downregulated PD-1, TOX, granzyme B, and Tim-3 since antigen is needed to sustain expression of these molecules but most interestingly, a subset of donor effector cells expressed *Tcf7*-YFP and CD127 in the absence of antigen. The co-staining of CD127, KLRG1, CD62L, and *Tcf7*-YFP (Extended Data Fig. 10c and d) shows that the PD-1+ Tim-3+ TOX+ GzmB+, and TCF-1 negative effector CD8+ T cell subset from day 5 clone 13 infection has the capacity to differentiate into the canonical short-lived effector cell (SLEC) subset (KLRG1+ CD127−) and more importantly also give rise to the memory precursor effector cell (MPEC) subset that expresses TCF-1 after clearance of antigen ^53-55^. This is consistent with our previous studies showing de-differentiation of a subset of effector CD8+ T cells after clearance of an acute viral infection^56-58^. A recent paper has further confirmed and extended these findings^59^. These previous studies examined effector CD8+ T cells generated during an acute infection and showed that the major pool of both effector and central memory cells emerges by the process of de-differentiation. We now show in this study that some of the effector CD8+ T cells generated during the early phase of a chronic infection also have the capacity to dedifferentiate and express TCF-1.

### Early day 5 virus-specific stem-like CD8+ T cells from LCMV Armstrong-infected mice act as chronic resource cells after transfer into chronically infected mice

**Figure 7:**
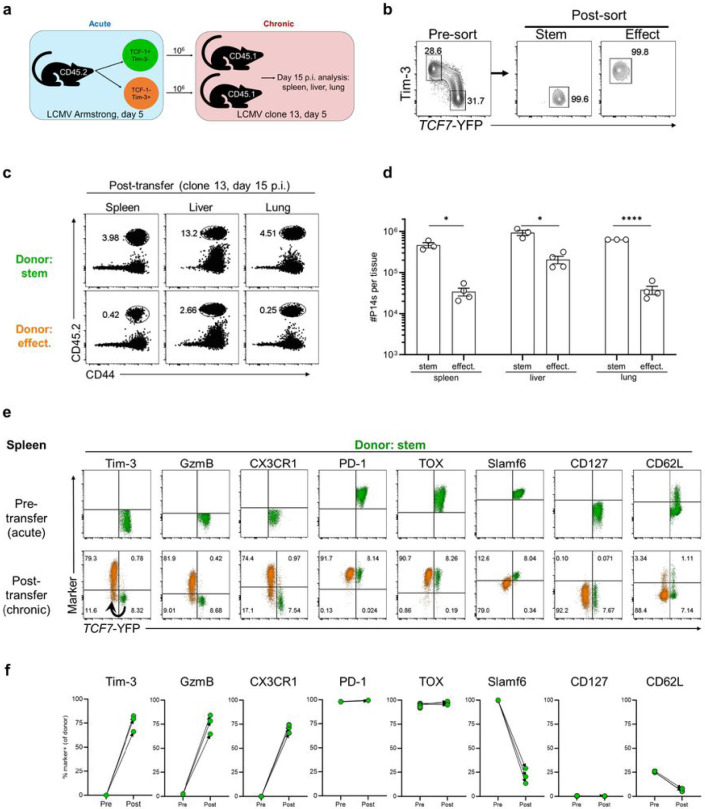
Early stem-like CD8+ T cells from acute viral infection become chronic resource CD8+ T cells after transfer into chronically infected mice. a,b, 2x10^3^ TCF7-YFP reporter P14 CD8+ T cells were transferred into congenically mismatched naïve mice prior to intravenous infection with 2x10^6^ PFU of LCMV Armstrong (acute). On day 5, TCF7-YFP+ Tim-3− (stem) and TCF7-YFP− Tim-3+ (effect.) P14 CD8+ T cells were FACS sorted from the spleen and 1x106 of each were transferred intravenously into separate groups of congenically distinct, day 5 LCMV clone 13 (chronic) mice. The recovery and phenotype of donor cells was analyzed 10 days later. c,d, Frequencies and numbers of stem-like and effector donor cells recovered from the spleens, livers, and lungs, of chronic LCMV recipient mice post-transfer. e, Expression of indicated markers on stem-like donor cells pre- (spleen, day 5 p.i., acute) and post-transfer (spleen, day 15 p.i., chronic). Arrow indicates differentiation trajectory of donor Tim-3- stem-like cells into Tim-3+ effector cells. f, Frequency of stem-like donor cells positive for indicated markers pre- and post-transfer in the spleen. Flow plots are representative of 1 independent experiment. Graphs show data from 1 independent experiment (pre-transfer n=9, post-transfer n= 3). Analysis of DbGP33+ TCF-1− Tim-3+ stem-like CD8+ T cells from a separate experiment was used to illustrate the pre-transfer expression of CX3CR1.

The studies described above showed that early day 5 stem-like CD8+ T cells generated during chronic infection can adapt to an acute infection setting and contribute to the pool of central memory cells (Fig. 6c, d, and Extended Fig. Data Fig. 10c). We now asked the reciprocal question of whether day 5 stem-like CD8+ T cells generated during an acute infection can act as resource cells in a chronic setting. The experimental design of this acute into chronic transfer is shown in Fig. 7a and b; sorted stem-like and effector P14 CD8+ T cells from LCMV Armstrong-infected mice at day 5 were transferred into mice infected with LCMV clone 13 at day 5. At day 15 post-clone 13 infection, the donor P14 cell populations were analyzed in the spleen, liver, and lung of recipient mice. We observed a significantly higher expansion of the transferred donor cells in all three tissues of mice that received day 5 P14 stem-like cells compared to P14 effectors (Fig. 7c and d). There were also striking changes in the phenotype of the donor stem-like cells in the spleen 10 days after transfer into chronically infected mice due to the generation of effector CD8+ T cells from the transferred donor stem-like cells (Fig. 7e and f). These progeny effector cells derived from the donor day 5 stem-like cells expressed effector markers such as granzyme B, CX3CR1, Tim-3, and downregulated stem-like markers *Tcf7*-YFP and Slamf6. These progeny effector cells comprised ~90% of the total P14 cells and the remaining ~10% retained the phenotype of the stem-like cells. This is important because it shows that the stem-like cells maintain themselves and also differentiate into effector cells. As expected, both the donor stem-like cells and their effector progeny expressed high levels of PD-1 and TOX. Similar results were seen in the liver and lung of mice that received the donor stem-like cells and in these non-lymphoid tissues the frequency of effector cells generated from the donor stem-like cells was even greater (>98%) (Extended Data Fig. 11a-d). Taken together these results show that day 5 P14 stem-like cells isolated from acute infection can function efficiently as a resource cell in the setting of a chronic infection.

In contrast to the efficient expansion and differentiation of the transferred P14 stem-like cells, the P14 effector cells showed significantly less expansion in all three tissues (spleen, liver, and lung) and showed minimal to no changes in their phenotypic markers (Fig. 7c, d, and Extended Data Fig. 12A-D). Even when five times fewer donor stem-like cells were transferred compared to donor effector cells, the stem-like cells gave rise to substantially higher numbers of progeny compared to effector donor cells (Extended Data Fig. 13a and b). Once again, these early (day 5) stem-like cells isolated from acutely infected mice differentiated into a large percentage of effector cells and also maintained their stem-like state to continue functioning as a resource cell under conditions of chronic infection (Extended Data Fig. 13c-f). This is a striking demonstration of how the host prepares to deal with a chronic infection very early, agnostic of the eventual outcome of the infection. The key chronic stem-like CD8+ T cells are already there to sustain T cell responses during chronic infection since without this cell the CD8+ T cell response would burn out.

In conclusion, this study provides new insight into the generation and maintenance of virus specific PD-1+ TCF-1+ TOX+ stem-like CD8+ T cells that are critical for maintaining the CD8+ T response during chronic infection. In particular, it highlights the early generation of these stem-like CD8+ T cells even before the outcome of infection is known and their abilty to adapt to an acute or chronic infection. These studies are of broad significance since the importance of these PD-1+ TCF-1+ stem-like CD8+ T cells has been documented not only in chronic viral infections but also in cancer and autoimmunity^60-63^.

## Methods

### Mice, viral infections, and virus titration

C57BL/6 and CD45.1 B6 mice were purchased from Jackson laboratory. CD45.1/45.1 or CD45.1/45.2 P14 TCR transgenic mice^64^ were bred and maintained in house. 6-8 week old mice were used for infections. *Nur77*-GFP transgenic mice were provided as a gift from the Au-Yeung laboratory at Emory University. Chronic viral infections that result in lifelong viremia were performed as follows: mice were transiently depleted of CD4+ T cells via intraperitoneal injection with 300 μg of a rat anti-mouse CD4 antibody (clone GK 1.5 from BioXcell) 2 days before infection and again on the day of infection. Mice were then injected intravenously (i.v.) with 2x10^6^ PFU LCMV clone 13 (Figs. 1, 5, and Extended Data Figs. 1-4, 5). All other figures feature chronic viral infections that eventually clear from most tissues. These were performed by injecting 2x10^6^ PFU LCMV clone 13 i.v. without transient CD4+ T cell depletion. For acute viral infections, mice were injected i.v. with 2x10^6^ PFU of LCMV Armstrong.

Quantification of infectious virus was performed via plaque assay on Vero E6 cells as previously described (Rafi/Oldstone, 1984 JEM paper). All mice were used in accordance with the Emory University Institutional Animal Care and Use Committee Guidelines.

### Lymphocyte isolation

Lymphocyte isolation from the blood, spleen, liver, and lungs were performed as described previously (Wherry’s JV paper). Spleens were dissociated by passing through a 70 uM cell strainer (Corning). Livers were first perfused with cold PBS prior homogenization via mechanical disruption. Lungs chopped prior to shaking at 37C in 1.3 mM EDTA in HBSS for 30 mins. They were then treated with 150 U mL^−1^ of collagenase (Thermo Fisher Scientific) in RPMI 1640 medium containing 5% FBS, 1mM MgCl_2_ and 1mM CaCl_2_ for 60 min at 37C with shaking at 200 rpm. Collogenase-treated lungs were then homogenized and filtered through a 70uM cell strainer. A 44-67% Percoll gradient was then used to purify lymphocytes (800g at 20C for 20min).

### Reagents and flow cytometry

All antibodies used for flow cytometry were purchased from Biolegend, Thermo Fisher Scientific, Miltenyi Biotec, Cell Signaling Technology, BD Biosciences, and R&D Systems. The following antibody/fluorochrome conjugates were used at the following dilutions: anti-CD8a PerCp-Cy5.5 (1:200), anti-CD8a BUV395 (1:200), anti-CD8b PerCp-Cy5.5 (1:200), anti-PD-1 Pe-Cy7 (1:200), anti-PD-1 BV785 (1:200), anti-CD44 Alexa Fluor 700 (1:200), anti-CD44 BUV737 (1:200), anti-CD4 BUV805 (1:200), anti-CD19 BUV805 (1:200), anti-B220 BV786 (1:200), anti-TCF-1 FITC (1:50), anti-Tim-3 PE (1:25), anti-Tim-3 FITC (1:25), anti-Granzyme B PE-CF594 (1:100), anti-CD73 APC (1:100), anti-CD73 BV421 (1:100), anti-CD28 PE (1:50), anti-CD28 APC (1:50), anti-Ki67 BV711 (1:25), anti-CD45.1 BV711 (1:200), anti-CD45.1 BUV563 (1:200), CD45.2 FITC (1:200), CD45.2 Pe-Cy7 (1:200), anti-TOX PE (1:50), anti-TOX APC (1:50), anti-BCL6 PE (1:50), anti-BCL6 APC (1:50), anti-SLAMF6 APC (1:100), anti-SLAMF6 Pacific blue (1:100), SLAMF6 BUV496 (1:100), anti-CD226 PE-Texas Red (1:100), anti-CX3CR1 PE-Cy7 (1:100), anti-CX3CR1 APC-Fire 810 (1:100), anti-KLRG1 PE/Dazzle 594 (1:100), anti-KLRG1 BV605 (1:100), anti-KLRG1 BV421 (1:100), anti-CD127 PE (1:50), anti-CD127 BV421 (1:50), anti-CD127 BV711 (1:50), anti-CD25 PE (1:100), anti-CXCR3 PE-Cy7 (1:100), anti-CD27 BV785 (1:100), and anti-CD62L BV605 (1:100). Endogneous LCMV-specific CD8 T cells were detected using the D^b^GP33-41 tetramer (1:100), which was prepared in house. Streptavidin-APC was purchased from (Thermo Fisher Scientific). Dead cells were excluded using the Live/Dead Fixable Near-IR (1:250) or Live/Dead Fixable Aqua (1:250) (Thermo Fisher Scientific). For cell surface staining, antibodies were prepared in PBS supplemented with 2% FBS at indicated concentrations before being added to cells on ice for 30mins. Cells were then washed two times. For detecting intracellular proteins (TCF-1, GzmB, TOX, and Bcl6), the FOXP3 staining buffer set (Thermo Fisher Scientific) was used according to the manufacturer’s instructions. Samples were acquired on a Canto, LSR II, the FAC Symphony A3 (BD Biosciences) system, or the Cytek Aurora spectral analyzer. Data were analyzed using FlowJo (v.10.7.1, BD Biosciences).

### Intravascular antibody labeling

For intravascular labeling, 3μg of BV421-conjugated anti-CD8α (Biolegend) was injected i.v. into infected mice. Three minutes after injection, splenocytes were isolated and prepared into a single cell suspension before staining *ex vivo.*

### *In vivo* peptide treatment

200ug of GP33 peptide was administered intravenously on days 5, 7, and 10 after acute LCMV infection (GenScript, RP20257).

### Cell Sorting

A FACS Aria II (BD Biosciences) was used for cell sorting. For experiments involving bulk RNA or ATAC sequencing, PD-1+ CD73+ Tim-3−, PD-1+ CD73− Tim-3+, PD-1+ CD44+ CD73+ Tim-3− P14s, PD-1+ CD44+ CD73− Tim-3+ P14s CD8 T cells from chronically infected (days 5 and >45 p.i.) or acutely infected mice (day 5 p.i.) were sorted at a purity of greater than 96%. This was true also for bulk RNA or ATAC sequencing of CD44+ PD-1− CD127+ KLRG1− and CD44+ PD-1− CD127− KLRG1+ P14 CD8 T cells from mice in which acute viral infection had resolved (day 15 p.i.). For experiments involving single cell RNA sequencing, PD-1+ GP33+ CD8 T cells from chronically infected (days 5 and >45 p.i.) or acutely infected mice (day 5 p.i.) were also sorted with greater than 96% purity. Naïve (CD44lo PD-1−) CD8 T cells were isolated from the spleens of uninfected mice. For experiments of adoptive transfer of stem-like and effector CD8 T cell subsets, 4000 naïve TCF7-YFP P14s were first transferred into uninfected mice followed by chronic LCMV infection. On day 5, splenocytes were isolated from chronic LCMV-infected mice (n=40-80) and 2x10^6^ to 6x10^6^ of two (CD8+ CD45.2+ YFP+ Tim-3− stem-like and CD8+ CD45.2+ YFP− Tim-3+ effector) subsets were sorted. The purities of sorted cells were greater than 95%.

### Adoptive transfers of stem-like and effector CD8 T cells

TCR transgenic P14 chimeras were generated by transferring splenocytes from naïve P14 mice (containing 2.5x10^3^ P14s) into congenically mismatched hosts one day prior to infection. For adoptive transfers assessing the function of day 5 subsets in response to LCMV clone 13 challenge, Slamf6+ Tim-3− stem-like or Slamf6− Tim-3+ effector P14 cells were FACs sorted from infected spleens of indicated donor mice. 2.5x10^3^ cells of each subset were transferred i.v. into naïve mice before recipients were infected 12 hours later with 2x10^6^ PFU LCMV clone 13 i.v. For adoptive transfers of sorted CD8 T cell subsets into infected, time-matched recipients, *TCF7*-YFP P14s (gifted by Hao Yuan Kueh) were first transferred into naïve congenically mismatched recipients prior to infection with 2x10^6^ PFU i.v. of LCMV Armstrong or clone 13. On day 5, *TCF7*-YFP+ Tim-3− stem-like or TCF7-YFP− Tim-3+ effector cells were FACs sorted from the spleens of infected mice and 1x10^6^ (or when indicated, 2.5x10^5^) cells of each were transferred into infected, time-matched recipients.

### Confocal microscopy of frozen spleen sections

To examine the localization of CD8+ T cell subsets in the spleen, spleens were removed from infected mice at indicated times and fixed overnight in periodate-lysine-paraformaldehyde buffer. Fixed spleens were then placed in 30% sucrose in PBS for 24 hours before embedding in optimal-cutting-temperature medium (Electron Microscopy Sciences) and freezing in dry-ice-cooled isopentane. 18 micron sections were cut on a Leica cryostat (Leica Microsystems). Sections were blocked with 1% bovine serum albumin (BSA), 5% mouse and 5% donkey serum in PBS wash containing 0.01% Tween 20 (Thermo Fisher Scientific) for 45-60mins at room temperature. Slides were then stained for Rat IgG2b anti-CD8β (1:200, Biolegend), Alexa 647 Rat IgG2a anti-PD-1 (clone 29F.1A12, 1:50, Biolegend), biotinylated Rat IgG2a B220 (1:200, Biolegend), and Rabbit IgG anti-TCF-1 (1:100, Cell Signaling) overnight at 4C in the dark. After washing, secondary antibody mix was added for 1 hour at room temperature in the dark containing Alexa 555 anti-Rat IgG2b (1:250, Southern Biotech), streptavidin-Alexa 594 (1:500, Thermo Fisher Scientific) and Alexa 488 anti-rabbit IgG (1:250, Thermo Fisher Scientific). LCMV antigen distribution was visualized on spleen sections, fixed and dehydrated as mentioned above, using guinea pig anti-LCMV sera (1:400) and Rat IgG2a anti-CD169 (1:200, clone 3D6.112, Biolegend). Following a 45-60min blocking step (1% BSA, 5% mouse serum, 5% goat serum, and mouse Fc block (Biolegend, 1:100) in PBS containing 0.01% Tween 20) and washing, sections were incubated with primary antibodies overnight at 4C in the dark. After washing with PBS containing 0.01% Tween 20, goat anti-guinea pig Alexa 555 or Alexa 647 (1:500, Thermo Fisher Scientific) and mouse anti-Rat IgG2A Alexa 488 (1:250, Southern Biotech) were added for 1hr at room temperature in the dark. After final washing steps, slides were mounted with Prolong Gold mounting medium containing DAPI (Thermo Fisher Scientific) and cover slipped. Images were acquired with a Leica SP8 tiling confocal microscope (Leica Microsystems) equipped with a 40x 1.3NA oil objective.

### Image analysis

Images were analyzed using Imaris software (Bitplane). Stem-like and effector/terminally differentiated CD8 T cells were identified accordingly:

1. The colocalization tool was used to create a new channel for PD-1+ CD8+ T cells.

2. 3D surfaces were then generated for PD-1+ CD8+ T cells to allow for detection of the DAPI nuclear stain, which was then used to distinguish cells from non-cells. A new channel was created for DAPI+ PD-1+ CD8+ T cells.

3. 3D surfaces were generated for DAPI+ PD-1+ CD8+ T cells to enable detection of TCF-1 stain. MFI of the TCF-1 channel was then determined for each surface and cutoff was set for TCF-1 positivity/negativity. Stem-like CD8 T cells = DAPI+ PD-1+ CD8+ TCF-1+. Effector/terminally diff. CD8 T cells = DAPI+ PD-1+ CD8+ TCF-1−. The white pulp and red pulp were manually annotated as discrete regions of interest (ROI) using the B220 and CD8β stains as a guide. Once total stem-like versus effector/terminally diff. 3D surfaces were distinguished from each other, the percentage of each located in the white or red pulp was calculated.

### scRNA-seq

scRNA-seq libraries were generated using the Chromium Single cell 5’ Library & Gel Bead Kit (10x Genomics) according to the manufacturer. D^b^GP33+ CD8 T cells or naïve CD44lo CD8 T cells were sorted and captured into gel beads-in-emulsion. After being reverse transcribed, gel beads-in-emulsion were disrupted and barcoded cDNA was isolated, pooled, then amplified by PCR for 13 cycles. Amplified cDNA was then fragmented and processed for end repair and A-tailing before undergoing sample index PCR for 16 cycles. Purified libraries were sequenced to a depth of 50,000 reads per cell on the HiSeq3000 (Illumina) systems with 26 cycles for read 1, 8 cycles for index (i7) and 91 cycles for read 2.

Cell Ranger v.3.1 was used to align, filter, and count barcodes as well as unique molecular identifiers. Further data analyses was performed using Seurat (v.3.0) (Satija, Nature biotech 2015). Cells with greater than 8% mitochondrial genes were excluded from analysis. Cells with more than 2,500-5,000 or less than 100-1,000 detected genes were considered outliers and excluded from downstream analyses. Raw unique molecular identifier counts were then normalized to unique molecular identifier counts per million total counts before being log-transformed. Principle component analysis was performed, and the 10 most statistically significant principal components were used for uniform manifold approximation and projection (UMAP) analysis. Clusters were identified using the nearest neighbor algorithm in Seurat and UMAP plots were generated based on selected PCA dimensions. The Seurat function FindAllMarkers was used to identify marker genes. Log-normalized data are shown. Gene set scoring was performed using the VISION R package v.2.1.0, following the scoring algorithm as described previously (DeTomaso, BMC Bioinformatics 2016). In brief, the expression of signature genes is weighted based on the predicted dropout probability calculated from nearest neighbors, and the normalized expression summed for all genes in the gene set. Gene set used: GSE84105 (Im et al., 2016).

### RNA seq

For experiments comparing CD8 T cell subsets on days 5 or >day 45 after acute or chronic LCMV infection, RNA was isolated from samples using the Qiagen RNA/DNA micro kit per the manufacturer’s instructions. Extracted RNA was sent to Emory Yerkes Nonhuman Primate Genomics core for mRNA library preparation and sequencing using the clontech SMART-Seq V4 and HiSeq1000. Data were normalized and differentially expressed genes were determined using DeSeq. A gene was considered differentially expressed with a normalized count >100, log_2_ fold-change >1 or <1 and adjusted P value <0.05. Data was analyzed using custom R scripts and visualized using ggplot2 R package, GraphPad Prism (v9.2), and excel. For Gene Set Enrichment Analysis (GSEA) each subset was analyzed using the pre-ranked list mode with 1,000 permutations. All gene sets used were from the MSigDB Molecule Signature Database unless otherwise noted. Enrichment scores were considered significant with an FDR <0.05 and was visualized using ggplot2 R package and GraphPad Prism (v9.2).

### ATAC seq

For experiments comparing CD8 T cell subsets on days 5 or >day 45 after acute or chronic LCMV infection, 2-4x10^3^ cells were sorted into PBS containing 2% FBS and transposition was performed as previously described (Guo, JI 2018 paper). Briefly, cells were resuspended in 12.5 μl 2x TD Buffer, 2.5 μl Tn5, 2.5 μl 1% Tween-20, 2.5 μl 0.2% Digitonin, and 5 μl H_2_O and incubated at 37C for 1 hr. Following transposition, cells were lysed with 2 μl 10 mg/ml Proteinase-K, 23 μl Tagmentation Clean-up buffer (326 mM NaCl, 109 mM EDTA, 0.63% SDS), and incubated at 40°C for 30 min. DNA was extracted and size-selected for small fragments using AMPureXP beads (Beckman Coulter, A63881) and PCR amplified into a sequencing library using NexteraXT indexing primers (Illumina, FC-131-2004) and KAPA HiFi HotStart Ready Mix (Roche, KK2602). Post-PCR final libraries underwent a second size selection using AMPureXP beads. Final ATAC-seq libraries were quantitated by qPCR and size distributions determined by bioanalyzer. Each sample was pooled at equimolar ratios and sequenced at the Emory Non-human Primate Genomics Core on a NovaSeq6000 using a PE100 run. Raw fastq reads were processed by removing adapter contamination with Skewer ^2^ and mapped to the mm10 genome using Bowtie2 v2.4.2. Accessible chromatin regions identified using MACS2 2.2.7.1 and differential accessibility between groups was determined using DESeq2 with a FDR < 0.05 and absolute log_2_ fold-change > 1. Transcription factor motif enrichment was assessed using chromVAR. Custom data analysis and data display was performed in R v4.1.0.

### Statistical analysis

GraphPad Prism (v.10.0.3) was used for statistical analysis. The difference between experimental groups was assessed using two-tailed unpaired t-tests or two-tailed unpaired Mann-Whitney *U*-tests or one-tailed paired Wilcoxon matched-pairs signed rank test.

## Data Availability

Custom code for scRNA-, RNA-, and ATAC-seq analyses can be provided from the corresponding author upon reasonable request.
